# Diffuse low-grade glioma misdiagnosed as acute cerebral infarction: A case report

**DOI:** 10.1097/MD.0000000000030378

**Published:** 2022-09-02

**Authors:** Yipo Ma, Jinfeng Zhang, Ying Wen, Jinghua Chen, Lei Yuan, Xuechun Jiang, Dan Xu, Kefu Liu

**Affiliations:** a Department of Radiology, Taicang City Hospital of Traditional Chinese Medicine, Taicang, Jiangsu, China; b Department of Neurology, Taicang City Hospital of Traditional Chinese Medicine, Taicang, Jiangsu, China; c Department of Cardiology, Taicang City Hospital of Traditional Chinese Medicine, Taicang, Jiangsu, China; d Department of Radiology, The Affiliated Suzhou Hospital of Nanjing Medical University, Suzhou, Jiangsu, China.

**Keywords:** case report, diffuse low-grade gliomas, ischemic stroke, olfactory hallucination

## Abstract

Diffuse low-grade gliomas (DLGGs) are relatively rare tumors that are more likely to be misdiagnosed and wrongly treated in clinical practice. We report a case of DLGG detected by computed tomography and magnetic resonance imaging (MRI).

**Patient concerns:**

A 58-year-old man suddenly phantom smells for half an hour and was previously healthy.

**Diagnoses:**

Computed tomography findings showed a leaf-shaped slightly hypodense shadow in the right temporal lobe with no obvious mass effect and an unclear boundary. MRI findings showed diffuse and slightly longer T1-weighted imaging (T1WI)/T2-weighted imaging (T2WI)signal in the right temporal lobe and hippocampus, slight hyperintensity on diffusion-weighted imaging, diffuse swelling in the right temporal lobe and hippocampus, and shallower cerebral sulci and fissures. No obvious abnormal enhancement was observed on enhanced MRI. Contrast-enhanced magnetic resonance angiography showed no obvious abnormality.

**Interventions:**

Intravenous thrombolysis with alteplase (rtPA) was given first.

**Outcomes:**

The patient had an acute and persistent generalized tonic-clonic seizure and was given antiepileptic treatment. Immunopathological and molecular genetic testing diagnosed as DLGGs. After targeted chemotherapy, the patient’s symptoms improved significantly.

**Lessons:**

For those cases with clinical acute neurological impairment and imaging findings similar to those of ischemic stroke, where the distribution of lesions is inconsistent with the distribution of blood vessels, and the time of onset does not match the imaging findings, the possibility of DLGGs should be considered.

## 1. Introduction

Diffuse low-grade gliomas (DLGGs) are relatively rare tumors in the nervous system, accounting for only 15% of gliomas.^[1]^ According to the 2021 World Health Organization classification,^[2]^ they mainly include diffuse astrocytomas and oligodendroglioma. The most common clinical manifestation of DLGGs is an epileptic seizure,^[3]^ and some cases showed neurological impairment associated with tissue invasion. Although histological examination and genotyping of tumors still serve as the golden standards for diagnosing DLGGs,^[4]^ such operations are relatively complex and have poor reproducibility, which are invasive and less acceptable among patients. Therefore, noninvasive imaging examinations are currently the preferred methods for diagnosing and evaluating DLGGs. However, imaging features of DLGGs are highly similar to those of ischemic stroke, making it difficult to give a diagnosis, so they are more likely to be misdiagnosed and wrongly treated in clinical practice. This paper reports a DLGGs case with olfactory hallucination as an initial symptom, which was misdiagnosed as acute cerebral infarction and treated with intravenous thrombolysis, leading to an epileptic seizure. Because of the retrospective analysis, oral informed consent was obtained from the patients for the publication of this case report and any accompanying images.

## 2. Case report

**Figure 1.**
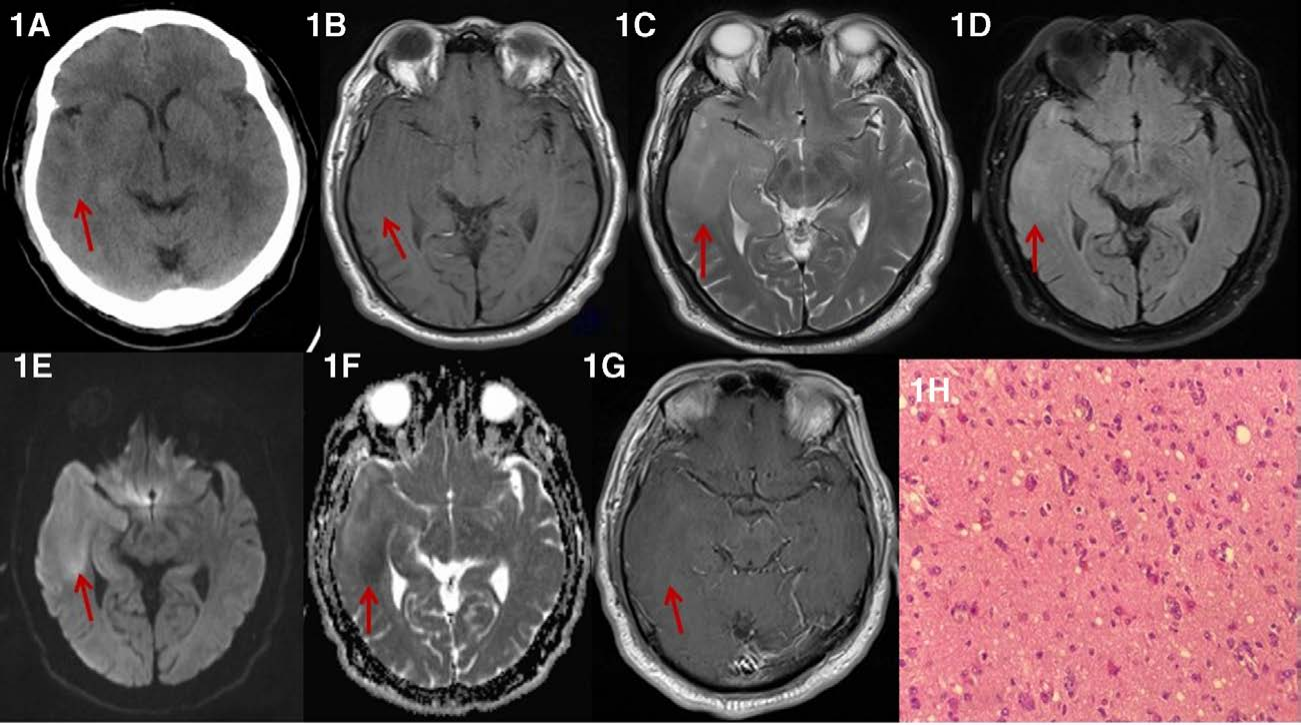
A 58-year-old man suddenly phantom smells for half an hour. (A) Head CT revealed a slightly hypodense in the right temporal lobe without obvious mass effect, which had unclear boundary; T1WI revealed slight hypointensity (B) and T2WI revealed slight hyperintensity (C)in the right temporal lobe and hippocampus; FLAIR revealed slight hyperintensity (D), DWI revealed slight hyperintensity (E) and ADC revealed slight hypointensity (F). (G) Enhanced examination showed no obvious enhancement, diffuse swelling in the right temporal lobe and hippocampus, and shallower cerebral sulci and fissures. (H) revealed diffuse proliferation of tumor cells with large and hyperchromatic nuclei, which was atypical, no necrosis or pathological karyokinesis was observed, and scattered lymphocytes were observed nearby (H&E ×400). ADC = apparent diffusion coefficient, CT = computed tomography, DWI = diffusion-weighted imaging, FLAIR = fluid attenuated inversion recovery, H&E = hematoxylin and eosin, T1WI = T1-weighted imaging, T2WI = T2-weighted imaging.

A 58-year-old man visited the emergency department at 12:30 due to “olfactory hallucination for half an hour.” The patient smelled irritating odors, mainly including rancid and fishy odors, which were obvious during exhalation and inhalation and persistent. Other people present on the scene smelled no abnormal odor. He was slightly irritable and had no visual abnormalities, impaired physical mobility, or convulsion. The patient received physical examinations, where the testing result of cranial nerves was negative, muscle strength and muscle tension of limbs were normal, pathological signs were negative, meningeal irritation signs were negative, and the results of routine blood test and related blood biochemistry tests were normal. At 12:45, the patient received head computed tomography (CT) scan (Fig. 1A), which showed a leaf-shaped slightly hypodense shadow in the right temporal lobe with no obvious mass effect and an unclear boundary. At 12:49, the patient’s muscle strength of the left limbs suddenly decreased (Grade V), and his condition was clinically diagnosed as acute cerebral infarction with a National Institute of Health Stroke Scale score of 0, so the patient was given intravenous thrombolysis with alteplase (rtPA) immediately (12:57) and admitted to the hospital. After thrombolysis, the symptoms did not improve significantly, and the patient received an emergency head magnetic resonance imaging (MRI) examination at 14:23 (Fig. 1B–F), showing diffuse and slightly longer T1-weighted imaging (T1WI)/T2-weighted imaging (T2WI) signal in the right temporal lobe and hippocampus, slight hyperintensity on diffusion-weighted imaging (DWI), diffuse swelling in the right temporal lobe and hippocampus, and shallower cerebral sulci and fissures. No obvious abnormal enhancement was observed in enhanced MRI (Fig. 1G). Contrast-enhanced magnetic resonance angiography (CE-MRA) of the head and neck showed no obvious abnormality. At 15:38, the patient had an acute and persistent generalized tonic-clonic seizure, and was given antiepileptic treatment. The corrected diagnosis considered the possibility of an intracranial tumor, so the patient was transferred to a higher-level hospital for stereotaxic biopsy of the right temporal lobe. Findings of the immunopathological examination (Fig. 1H): *glial fibrillary acid protein* (+), Vimentin (+), olig-2 (partially +), S-100 (+), *alpha thalassemia/mental retardation syndrome X-linked* (+), *cytokeratin pan* (–), *isocitrate dehydrogenase (IDH*) (–), and Ki-67 (30%–40%+); diffuse astrocytoma in the right temporal lobe, *IDH* wild-type, not excluding glioblastoma. Findings of molecular genetic testing: no Chr1P/19q co-deletion, *telomerase reverse transcriptase* promoter-mutation glioblastoma, World Health Organization Class-IV, negative methylation in the *O-6-methylguanine-DNA methyltransferase* promoter region, pathogenic mutations detected in the *PIK3CA* and *NF1* genes, deletion variant in the *CDKN2A* gene, MDM4 copy number amplification, and no germline mutations associated with hereditary brain tumors. After targeted chemotherapy and other treatments, the patient’s symptoms improved significantly.

## 3. Discussion

Epilepsy is an initial symptom of most DLGG patients. It was reported that the incidence of olfactory abnormalities increased by 76% after the occurrence of a stroke,^[5]^ olfactory abnormalities may occur in untreated solid tumors and after radiotherapy or chemotherapy, especially in glioblastoma, which is related to olfactory receptors. In this case, the lesions in the patient were located in the right temporal lobe and hippocampus, involving the olfactory center,^[6]^ so attention should be paid to olfactory abnormalities.

This patient has been healthy before the onset, who had an acute onset and neurological impairment manifested by persistent olfactory hallucination. The limited low-density foci in the right temporal lobe observed during the head CT scan were in line with the location of the olfactory center, and there was no bleeding or space-occupying. Then the patient had reduced muscle strength and thus was clinically diagnosed with acute ischemic stroke. According to the rules on diagnosis and treatment specified in relevant guidelines,^[7]^ there were indications for intravenous thrombolysis, so the patient was given a standard dose of rtPA (0.9 mg/kg) for thrombolysis. Although early hypodense signs appeared during the head CT scan, the patient had definite time of onset and neurological impairment. In addition, subsequent MR images showed hypointensity on T1WI, limited DWI, and hyperintensity on fluid-attenuated inversion recovery (FLAIR) or T2WI, so DWI did not match FLAIR, and intravenous thrombolysis still brought clinical benefits.

This patient developed a persistent generalized tonic-clonic seizure after intravenous thrombolysis. Was it post–stroke epilepsy? Was it related to intravenous thrombolysis? Was olfactory hallucination a premonitory symptom of epilepsy? We systematically reviewed relevant literature and found that epilepsy is related to the severity, etiology and location of a stroke,^[8]^ and the incidence of status epilepticus is 16.3%, which is more common in cortical infarction or cerebral infarction caused by main artery occlusion, or occurs in patients who have a history of stroke, and may be accompanied by negative neurological symptoms.^[9]^ In this case, the patient’s medical history and lesion location were not in line with the above characteristics. Diffuse gliomas with IDH mutations feature a higher incidence of epilepsy, which is often an initial clinical symptom, and can also lead to manifestations of ischemic stroke^[10]^; however, intracranial tumors are still absolute contraindications to intravenous thrombolysis because reperfusion injury is associated with a higher risk of symptomatic intracranial hemorrhage, and it cannot improve the neurological improvement rate and long-term survival benefits.^[11]^ Most clinical evidences show that the use of intravenous thrombolysis and mechanical thrombectomy does not increase the risk of post–stroke epilepsy, and post-thrombolysis hemorrhagic transformation is an independent risk factor for post–stroke epilepsy or epilepsy.^[12]^ In this case, no evidence of bleeding was found during the CT scan, and CE-MRA showed no large vessel occlusion during reexamination, which did not support the hypothesis of post-thrombolysis hemorrhagic transformation or epilepsy after massive stroke. As a common symptom of temporal lobe epilepsy, olfactory disorder is manifested by hyposmia or anosmia in most cases. This case was manifested by olfactory hallucination, which was nearly 4 hours away from status epilepticus, and was different from premonitory symptoms of epilepsy in terms of the form of onset.^[13]^ Therefore, we believe that the epileptic seizure in this patient was related to the intracranial tumor receiving intravenous thrombolysis, where the mechanism might be abnormal expansion of blood vessels in the tumor and excessive tissue perfusion.

In this case, the patient had an acute onset, where the olfactory hallucination was related to lesions in DLGGs in the temporal lobe, and in line with the localization of the olfactory center. The head CT showed an early low-density shadow related to the localization of stroke symptoms, and the patient had reduced muscle strength in the contralateral limbs, which was one of the causes of misdiagnosis. In addition, this patient’s MRI showed hypointensity on T1WI, hyperintensity on FLAIR or T2WI, and no enhancement on MRI, which were very similar to the imaging findings of ischemic stroke. This was another cause of misdiagnosis. MRI showed that the lesions were large in scope and involved multiple cerebrovascular blood supply areas, but CE-MRA showed no abnormal large blood vessels, so the imaging lesions were not in line with the responsible blood vessels, which was also a major distinguishing feature of DLGGs. Grass-roots doctors and some young clinicians are likely to misdiagnose them as ischemic stroke due to lack of experience or knowledge. As far as we know, DLGGs are very likely to be misdiagnosed as cerebrovascular diseases, central nervous system infections or demyelinating disorders. This is the report of the first DLGG case where olfactory hallucination and olfactory hyperesthesia were initial symptoms, which was misdiagnosed as acute ischemic cerebral infarction and treated with intravenous thrombolysis, inducing a generalized tonic-clonic seizure.

In conclusion, for those cases with clinical acute neurological impairment and imaging findings similar to those of ischemic stroke, where the distribution of lesions is inconsistent with the distribution of blood vessels, and the time of onset does not match the imaging findings, the possibility of DLGGs should be considered. It is necessary to be cautious about clinical treatment to prevent misdiagnosis and wrong treatment, and the final diagnosis depends on pathological biopsy.

## Author contributions

Validation: Kefu Liu

Visualization: Xuechun Jiang, Dan Xu, Ying Wen, Lei Yuan

Writing - original draft: Yipo Ma, Jinfeng Zhang

Writing- review & editing: Jinghua Chen, Kefu Liu
